# Epigenomic Regulation of Androgen Receptor Signaling: Potential Role in Prostate Cancer Therapy

**DOI:** 10.3390/cancers9010009

**Published:** 2017-01-16

**Authors:** Vito Cucchiara, Joy C. Yang, Vincenzo Mirone, Allen C. Gao, Michael G. Rosenfeld, Christopher P. Evans

**Affiliations:** 1 Department of Urology, School of Medicine, University of California, Davis, 4860 Y Street, Suite 3500, Sacramento, CA 95817, USA; vito.cucchiara89@gmail.com (V.C.); jcyang@ucdavis.edu (J.C.Y.); acgao@ucdavis.edu (A.C.G.); 2 Department of Neurosciences, Reproductive Sciences and Odontostomatology, University Federico II, Naples 80131, Italy; mirone@unina.it; 3 Department of Medicine, Howard Hughes Medical Institute, University of California San Diego, La Jolla, CA 92093, USA; mrosenfeld@ucsd.edu; 4 Comprehensive Cancer Center, UC Davis School of Medicine, University of California, Davis, Sacramento, CA 95817, USA

**Keywords:** epigenetics, prostate cancer, androgen receptor, methylation, acetylation, non-coding RNA, biomarkers, novel treatments

## Abstract

Androgen receptor (AR) signaling remains the major oncogenic pathway in prostate cancer (PCa). Androgen-deprivation therapy (ADT) is the principle treatment for locally advanced and metastatic disease. However, a significant number of patients acquire treatment resistance leading to castration resistant prostate cancer (CRPC). Epigenetics, the study of heritable and reversible changes in gene expression without alterations in DNA sequences, is a crucial regulatory step in AR signaling. We and others, recently described the technological advance Chem-seq, a method to identify the interaction between a drug and the genome. This has permitted better understanding of the underlying regulatory mechanisms of AR during carcinogenesis and revealed the importance of epigenetic modifiers. In screening for new epigenomic modifiying drugs, we identified SD-70, and found that this demethylase inhibitor is effective in CRPC cells in combination with current therapies. The aim of this review is to explore the role of epigenetic modifications as biomarkers for detection, prognosis, and risk evaluation of PCa. Furthermore, we also provide an update of the recent findings on the epigenetic key processes (DNA methylation, chromatin modifications and alterations in noncoding RNA profiles) involved in AR expression and their possible role as therapeutic targets.

## 1. Introduction

Prostate cancer (PCa) is the most prevalent cancer in men and the third cause of cancer-specific mortality in Western countries [1]. To understand the cornerstone of prostate carcinogenesis, many authors have pointed towards the central role of the androgen receptor (AR). AR, a member of the nuclear receptor superfamily and located at chromosome Xq11-12, contains three major functional domains. The first, highly unstructured, and largest domain is the N-terminal domain (NTD), which comprises the activation function 1 (AF1) motif. The DNA binding domain (DBD) is the second AR-region and contains two zinc fingers that cooperate with the androgen-response element (ARE), and allow dimerization. The hinge region is a bridge between the DBD and the ligand binding domain (LBD), which accommodates the second activation function (AF2) motif [2]. It is well established that sustained AR activity is inexorable from PCa cell survival and disease progression, even following androgen deprivation therapy (ADT) [3,4]. Since the discovery in the 1940s that PCa is dependent on androgens [5], the central therapy for patients with locally advanced or metastatic disease targets the AR. After an initial period of therapeutic response, PCa become insensitive to these therapies and progresses to the castration resistant prostate cancer (CRPC) [6]. To date, in addition to the well- known genetic mutations, epigenetics is considered fundamental in the molecular pathogenesis of PCa. Epigenetics has been described as “the stable transmission of cellular information due to a modification of the DNA without a change in DNA sequence” [7,8]. It has been demonstrated that alteration of epigenetic marks may determine cancer initiation, development, and subsequent progression [9,10]. This review focuses on the role of epigenetic processes such as histone methylation, histone acetylation and non-coding RNA that play a central role in the regulation of AR in PCa pathogenesis and progression and discusses further modalities of treatment.

## 2. Histone Methylation

Histone methylation is an important and complex method of transcriptional control mediated by histone methyltransferase (HMT) and histone demethylase (HDM) enzymes. Methylation changes to the local chromatin encourage or repress transcription according to the site of modification [11]. For example, methylation of lysine residues 4 and 36 in histone H3 (H3K4, H3K36) generally preserves euchromatic domains [12,13] whereas the modification of H3K9 and H3K27 [14,15] forms heterochromatic regions. Arginine methylation is an alternative method of histone modification. Protein arginine methyltransferase (PRMT) family members such as PRMT6 [16] and coactivator associated arginine methyltransferase 1 (CARM1) [17], are enzymes responsible for histone methylation at arginine residues. Several articles [2,18] suggest that the histone methylation of AR can regulate the transcriptional activity of AR.

One of the most extensively studied HMT enzymes in PCa is SET9, which seems to improve gene expression by inducing histone H3K4me1 and obstructing histone H3K9 methylation and the nucleosome remodeling deacetylase (NURD) complex [19,20,21]. Different groups have observed elevated levels of this enzyme in malignant epithelial cells from PCa patients [22,23]. To explore its role in the regulation of the AR, many works describe that SET9 is responsible for N–C inter-domain cooperation that is important for AR transcriptional activity [24,25,26]. It was subsequently found that the hinge region of AR contains a motif (KLKK) that is comparable to the sites modified by the methyltransferase SET9 in other proteins [22,23]. Even if SET9 was shown to methylate AR, a consensus could still remain elusive about the sensitivity of this interaction. It is also unclear which Lys is methylated; one study shows Lys 630 [23] and another Lys 632 [22].

The nuclear receptor-binding SET domain-protein 2 (NSD2) is a histone methyltransferase that cooperates with the DBD of the AR [27]. High levels of NSD2 are related to the expression of PSA (prostate specific antigen) [27]. A paper by Asangani et al. reported that high levels of NSD2 correlate with aggressive characteristics in PCa [28]. The mechanism of action is linked to the enhancer of zeste homolog 2 (EZH2), a component of Polycomb repressive complex 2 (PRC2) [4]. The enhancement of EZH2 leads to the transcriptional inhibition of miR-203, miR-31 and miR-26, which are repressors of NSD2. This complex mechanism facilitates an over expression of NSD2 with the generation of the active histone mark, H3K36me2. Moreover, the study by Yang et al. [29] shows that NSD2 acts as a transcriptional coactivator of NF-κB for activation of target genes, such as *IL-6*, *IL-8*, *VEGFA* and *survivin* in CRPC cells.

Historically, EZH2 has been considered an AR transcriptional repressor. This peculiarity has been related to the ability of EZH2 to catalyze two repressive histone markers, H3K27me3 and H3K4me3, via AR recruitment [30]. Other works with conflicting findings have established a strong correlation between increased EZH2 and more aggressive [31], neuroendocrine [32] or metastatic [33] PCa. The role of EZH2 as an AR coactivator has been described to be AKT dependent. In fact, the phosphorylation of EZH2 serine 21 mediated by PI3K/AKT obstructs the methylation of H3K27 [34]. Xu et al. [35] confirmed these previous reports and showed that the phosphorylation of EZH2 at serine 21 defines the oncogenic function of EZH2 as a coactivator of AR in advanced PCa. This mechanism is independent of PCRC2 and H3K27me3 and suggests that EZH2 can methylate other proteins or other histone residues.

Another methyltransferase involved in PCa growth is PRMT6 [36]. PRMT6 has a high affinity for H3 and provides H3R2me2, a well-known repressive mark [36] but at the same time it was widely detected in a cohort of patients affected by PCa [37]. Almeida-Rios et al. [38] recently showed that PRMT6 silencing in PC-3 cells downregulates the PI3K/AKT/mTOR pathway and increases AR signaling.

A relevant enzyme for the AR regulation is the lysine specific demethylase 1 (LSD1). It has been targeted for its dual ability to suppress or stimulate AR expression [18]. The explanation of its role as a transcriptional coactivator can be the de-methylation of H3K9me1,2 [39]. The activity of this methyltransferase could be regulated by other post-transcriptional modifications. For example, it was discovered that H3 phosphorylation mediated by the protein kinase C-related kinase 1 (PRK1) [40] and the protein kinase C 1 (PKC1) [41] changes the substrate of LSD1 from H3K4me1,2 to H3K9me1,2 with an enhancement of AR related gene expression. Recently, Yang et al. [42] described an alternative mechanism of LSD1 that involves the generation of ROS leading to DNA damage. The authors report that this ROS generation occurs after androgen stimulation, which determines the demethylation of H3K4me1,2 on ARE regions, resulting in DNA damage. This DNA damage releases DNA and facilitates DNA loop formation, which is critical for miRNA expression and transcription. Subsequently, OGG1 and APEX1, DNA damage repair factors, are recruited to these ARE regions in an androgen and LSD1 dependent manner, suggesting that LSD1-mediated AR targets transcription relies on H3K4 demethylation and DNA oxidation [42].

Historically, despite its aforementioned role as coactivator, LSD1 has been considered a corepressor. LSD1 acts as a demethylase for H3K4me1,2 [43] enhancing the recruitment of corepressor complexes. Moreover, has been reported that LSD1 can reduce the expression of several genes such as the AR gene or *AKR1C3* and *HSD17B6*, two genes responsible for the androgen synthesis [18,44]. The overexpression of AKR1C3 have been correlated with PCa progression and aggressiveness [45,46] and recent findings describe the activation of AKR1C3 as a mechanism of resistance to Enzalutamide and Abiraterone [47,48].

Furthermore, it has been shown that other HMT enzymes such as the lysine demethylase 4B (KDM4B) [49], KDM4C [50], and KDM3A [51] can enhance the AR transcription activity. KDM4B, an enzyme that can de-methylate H3K9me3, has a duplex function. It can stimulate the AR activity directly through the demethylation of H3K9me3, or indirectly reducing the ubiquitylation and degradation of AR [49].

It is well known that one of the tumorigenic mechanisms in PCa cells is the fusion gene *TMPRSS2-ERG* [52] and several works highlight the causal relationship between the AR signaling and these genomic rearrangements [53]. Androgen stimulation facilitates the co-recruitment of the AR and the topoisomerase II beta (TOP2B) at *TMPRSS2* and *ERG* loci near genomic breakpoints, leading to TOP2B-mediated DNA double strand break formation [54].

**Figure 1:**
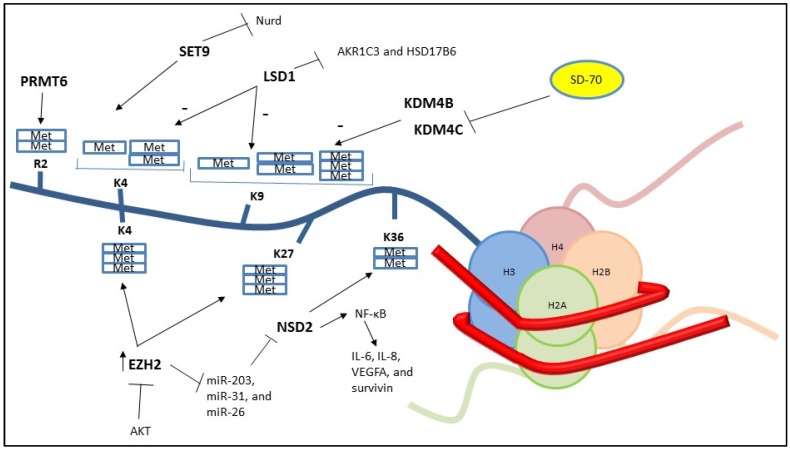
Schematic overview of AR histone 3 methylation status. SD70 inhibits the demethylase activity of KDM4C and is effective in CRPC cells both in vitro and in vivo. NURD: nucleosome remodeling deacetylase complex; EZH2: enhancer of zeste homolog 2; LSD1: lysine specific demethylase 1; NSD2: nuclear receptor-binding SET domain-protein 2; PRMT6: protein arginine methyltransferase 6; KDM4B and KDM4C: Lysine Demethylase 4B and 4C.

Yu et al. [55], through the use of a chromatin precipitation (ChIP) technique, discovered that ERG expression increases the recruitment of EZH2 which may then mediate the repression of AR transcription activity through H3K27 methylation [55]. Using a global proteomics approach to unravel the mechanism that might control androgen-dependent *TMPRSS2-ERG* fusion, Metzeger et al. [56] showed that the di-methylation of K114 mediated by LSD1 is executed by the histone mehylatransferase EHMT2. LSD1-K114me2 allows for interactions with the chromodomain helicase DNA-binding protein 1 (CHD1). The complex (EHMT2-LSD1 K114me2-CHD1) controls chromatin binding of AR, and it was found to play an important role in regulating the *TMPRSS2-ERG* oncogenic fusion [56]. The mechanisms of action of the principal methyltransferases and demethylases involved in the regulation of AR gene expression are presented in Figure 1.

## 3. Histone Acetylation

The histone acetyltransferases (HAT) and histone deacetylases (HDAC) are two groups of enzymes that regulate acetylation and deacetylation [57]. In general, active euchromatin is relatively hyperacetylated whereas inactive heterochromatin is hypoacetylated [58]. In 2000, Fu et al. discovered within the flexible hinge region of AR a short sequence (KLKK), with the property of an acetylation motif [59].

As described in other reviews [18,60,61,62], the histone acetylation status is a reversible process of placing and removing covalent acetyl groups that can improve or reduce the AR transcriptional activity, respectively. To study the fundamental role of AR acetylation, several groups used two different models in which the acetyl acceptor sites were mutated to be non-functional or acetylation mimetic. In these two scenarios, when the AR is non-functional, the AR takes on a repressed form, which increases binding to co-repressor proteins including NCoR [59,63,64]. In the other case, when the acetylation acceptor mutated sites mimic acetylation, we can observe a completely different result; an improvement of the transcriptional activity and a reduction of the interaction with co-repressor proteins [60,63].

### 3.1. AR Activation Mediated by Histone Acetylation

Many works have described several co-regulators of the AR transcription machinery with a HAT activity such as p300/CAF [59], p160/SRC [65], Tat-interactive protein, 60 kDa (TIP60) [66], and *N*-acetyltransferase arrest-defect 1 protein (ARD1) [67].

CBP and p300 are proteins with HAT activity, and they are able to regulate transcription [68,69]. It was discovered that AR is acetylated by p300 and p300/cAMP-response element-binding protein associated factor (PCAF) both in vitro and in vivo [59]. Recently, Zhong et al. [70] explained an interesting pathway involving PTEN and AKT. The authors show that the inactivation or deletion of PTEN and the subsequent phosphorylation of AR at the serine 81 stimulates the acetylation of the AR by p300. Furthermore, it has been described that p300 can affect the AR activity indirectly. In fact, the acetylation of b-catenin provided by p300, determines a different interaction with the AR leading to an enhanced AR transcription [71].

The Steroid Receptor Coactivator-1 (SRC1) is responsible for the activation of AR due to its HAT domain [72]. Moreover, it has not only been shown that SRC1 can interact directly with AR, but it can recruit other coactivators (p300/CBP) in order to stimulate the transcriptional activity of the AR [73]. A recent study describes the possible role of the SCR1/p160 binding site as a novel therapeutic target. In fact, using two overlapping SRC1 peptides the authors show an inhibition of AR-dependent genes, such as *PSA* and *TMPRSS2* [74].

As previously suggested in another review [60], in addition to androgens, various other factors can stimulate the levels of AR acetylation mediated by CBP/p300 or SRC1. Despite the fact that the mechanism of action is not well-understood, it has been proposed that bombesin, via Src and PKC signaling pathways, can activate p300 activity. This interaction leads to enhanced acetylation of AR resulting in increased expression of AR-regulated genes (*PSA*) [75]. At the same time, IL-4 increases CBP/p300 protein expression and enhances interaction of AR with CBP/p300 proteins through a recruitment of p300 protein to the androgen responsive elements (AREs) in the promoters of androgen responsive genes [76]. IL-6 is another cytokine important for cell growth and survival in PCa both in vitro and in vivo [77], and it has been reported that SRC-1 can improve its ligand independent stimulation of AR by IL-6 via MAPK [78].

TIP60, an AR factor acetyl transferase (FAT), has a specificity for the LBD of the AR [79]. More recently, it has been shown that TIP60 may be directly responsible for the acetylation of AR and it can interplay with HDACs at the PSA promoter gene. The equilibrium between these can lead to activation or suppression of AR transcription [80]. Shiota et al. [81] explained that TIP60 overexpression facilitates the acetylated form of AR and, consequently, the AR localization in the nucleus in absence of an androgen enriched environment.

Arrest defective-1 protein (ARD1) is another acetyltransferase [82] which has important functions in several types of cancer through acetylating different target proteins [83,84,85]. Wang et al. [67] reported that the level of ARD1 is consistently higher in PCa, and recently, a work by DePaolo et al. [86] revealed that ARD1 not only acetylates AR at lysine 618 but also creates a ternary complex with AR and HSP90, playing a role in the AR-HS90 dissociation.

Interestingly, another study suggests that the levels of AR potentiate the recruitment of AR and the components of the transcription machinery to chromatin in order to enhance the acetylation on H3K9 and on H3K14 in CRPC cells even in an androgen deprivation environment [87]. These finding are in line with other works [88], which report how an enhanced acetylation in cells that overexpress AR is linked to the development of a castration resistant condition.

### 3.2. AR Inhibition Mediated by Histone Acetylation

As mentioned above, acetylation of particular residues determines the enhancement of the AR activity, and it is normal to expect that the opposite process can lead to inhibition. Within the HDAC family, we encounter several proteins with a similar enzymatic activity. For example, HDAC1 interacts with the PSA promoter and suppresses AR signaling [66] while HDAC7 has the similar ability to inhibit AR, but in this case the mechanism of action is independent of AR acetyl acceptor sites [89]. Moreover, several studies describe that HDAC6 regulates the correct folding of the AR mainly via modulating HSP90 acetylation. The acetylation of HSP90 results in a destabilization of the AR and subsequently in its degradation by the proteasome [90].

**Figure 2:**
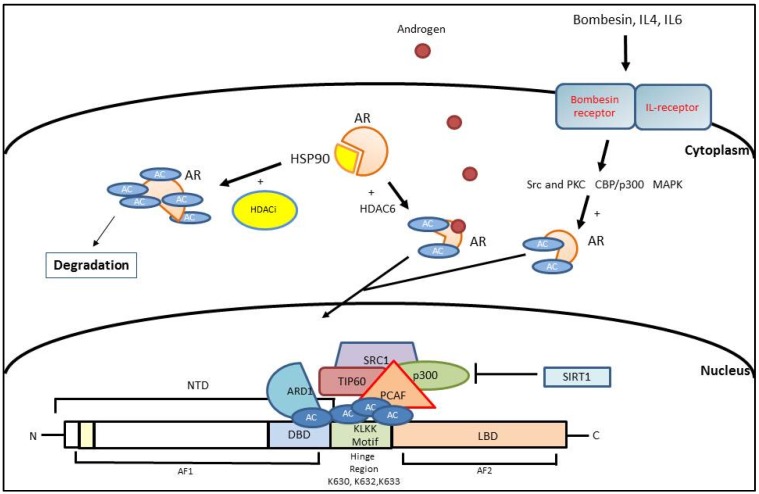
Graphic representation of the balance between acetylation and deacetylation in the regulation of androgen receptor (AR) gene expression. The mechanism of action of histone deacetylase inhibitors (HDACi) such as romidepsin and panobinostat is related to the heat shock protein-90 (HSP90). NTD: N-terminal domain; DBD: DNA binding domain; LBD: ligand binding domain; AF1 and AF2: activation function 1 and 2; PKC: protein kinase C; SRC1: steroid receptor coactivator-1; TIP60: Tat-interactive protein, 60 kDa; ARD1: *N*-acetyltransferase arrest-defect 1; SIRT1: Sirtuin 1; PCAF: p300/cAMP-response element-binding protein associated factor.

Sirtuin 1 (SIRT1), a NAD-dependent deacetylase, has been described as a repressor of AR activity [91]. Fu et al. extended precious observations and established a role for SIRT1 in regulating cellular growth by repressing and deacetylating the AR directly [91]. Moreover, the same group highlighted a “functional antagonism” between SIRT1 and p300 at the same site of the hinge region avoiding the N-C terminal interaction [61,91]. Figure 2 depicts the molecular communication between AR and acetylation status in order to enhance or reduce AR gene expression.

## 4. Non-Coding RNA

In the last decade, several articles corroborated by the use of new technologies to reveal that a major portion of the non-coding genome is transcribed with many regulatory functions. This brought about a change in thinking that non-coding RNA can have a role in cancer [92]. Non-coding RNAs (ncRNAs) are divided into two major groups based on their size: small ncRNA (<200 bp) and long ncRNA (>200 bp) [93].

### 4.1. MicroRNA and AR

MicroRNAs (miRNAs) are a class of small non-coding RNAs with an important role in cell development, differentiation and signal transduction. Generally, miRNAs cause mRNA translational repression or mRNA degradation by binding to the 3′ untranslated region (3′-UTR) [94]. Furthermore, recent studies have reported that the 5′-UTR of mRNAs might be involved in the gene regulation by miRNA, and it has been shown that miRNA can activate gene expression rather than repress it [95,96]. Based on the central role of AR signaling in the normal and neoplastic growth of the prostate cell, many reports describe the existence of feedback loops between miRNAs and AR [97].

#### 4.1.1. Androgen Regulation of miRNA Expression

In 2011, Waltering et al. presented one of the first miR microarray studies to examine androgen regulation of miRNAs [98], and they showed that dihydrotestosterone (DHT) positively modulates 17 miRNAs in VCaP cells whereas castration causes high levels of 42 miRNAs. The work of several independent groups demonstrates that miRNAs such as miR-19a, miR-148, and miR-27a are androgen inducible miRNAs [99,100,101]. Indeed, androgen-mediated overexpression of miR-27a results in the reduction of prohibitin, a well-known tumor-suppressor gene and co-repressor of the AR, with a subsequently increased expression of AR genes and increased PCa cell growth [101].

Genome-wide screenings of androgen target genes have identified miR-125b as androgen-inducible miRNA [102] and in particular have been shown that androgens carry out this action by binding the promoter region of the miR-125b gene. Moreover, Sun et al. [103] reported that AR targets the miR-99a/let7c/125b-2 cluster genes region LNC00478 and subsequently represses the level of this cluster. The authors also explain the role of two chromatin modifiers EZH2 or JMJD3, that can suppress or enhance the levels of the miR-99a/let7c/125b-2 cluster depending on the presence or the absence of androgen [103]. The downregulation of the miR-99a/let7c/125b-2 cluster has been shown to protect many of their target mRNAs from degradation. On the contrary, when miR-125b is overexpressed, it cooperates with the insulin-like growth factor 1 (IGF1R) to enhance PCa cell development [103]. MiR-125b has been reported to stimulate the PCa cells growth without androgen stimulation through down-regulating the expression of Bak1 (Bcl-2 homologous antagonist/killer 1) [104] and by targeting the Bcl-2-binding component 3 (BBC3) and p53 [105,106,107]. MiR-125b, as described in another work [108], is connected to Her2-AR pathway and could have a function in inducing CRPC.

MiR-135a has been found to be upregulated in androgen sensitive PCa cells and AR, as previously reported for miR-125, directly activates transcription by using a functional ARE in the miR-135a promoter region [109]. To explore the biological effects of miR-135a in prostate cells, the researchers overexpressed miR-135a in LNCaP cells and demonstrated that miR-135a can down-regulate the expression of the Rho-associated protein kinase 1 (*ROCK1*) and *ROCK2* (implicated in cytoskeleton regulation) at mRNA and protein levels [109]. Coarfa et al. [110] also found AR recruitment to the ARE in the promoter region under androgen stimulation. They additionally identified stronger co-recruitment of AR and coactivators to a region immediately downstream of the miR-135a-5p gene without the addition of androgen. Combined with the inhibitory effect of miR-135a-5p on expression of AR and its coactivators, this suggests a negative feedback loop that can de-repress AR axis transcriptional output upon androgen deprivation. A recent study by Wan et al. [111] describes a downregulation of miR-135a in CRPC. The authors found that RB-associated KRAB zinc finger (*RBAK*) and matrix metalloproteinase 11 (*MMP11*), two genes involved in migration pathway, are controlled by miR-135a. They showed that PCa progression is associated with low levels of miR-135a and high levels of RBAK and MMP11.

MiR-32 is also reportedly an androgen-regulated miRNA. The transfection of pre-miR-32 into LNCaP cells confers significant cell growth and reduces apoptosis. In CRPC, miR-32 is regulated by androgen through targeting the B-cell translocation gene 2 (*BTG2*), a member of the antiproliferative (APRO) gene family [112]. BTG2 regulates several cellular mechanisms such as cell cycle progression, DNA damage repair, and apoptosis, and thus it has been shown that its levels are suppressed in many human cancers [113].

AR acts as a stimulus for miR-21 transcription by targeting miPPR-21, the miR-21 promoter [114]. AR is not the only enhancer of miR-21. In fact, mir-21 can be stimulated by two other transcriptional factors, the activator protein 1 (AP-1) and the signal transducer and activator of transcription 3 (STAT3) [115,116]. Furthermore, Mishra et al. [117] described a positive feedback loop between miR-21 and AR. The AR and miR-21 axis negatively alters the TGFBR2 pathway, and in this way inhibits the tumor-suppressive activity of TGFβ. Mir-21 is implicated even in the regulation of the cell cycle, and the same group further revealed that miR-21 is not only able to reduce the level of a cyclin-dependent kinase inhibitor p57Kip2, but it is also able to attenuate p57Kip2-mediated responses [118].

MiR-221 and miR-222 are encoded on the X chromosome [119], but curiously they are downregulated by AR in an androgen enriched environment [112]. A recent review by Shih et al. [97] highlighted the mutual interaction between miR-221 and AR. Even though miR-221 has been extensively studied, we still do not have a clear idea on what its expression pattern in PCa is. For example, work by Gordanpour et al. [120] shows low levels of miR-221 in aggressive PCa with an inverse association with the Gleason Score, clinical recurrence, and metastasis. On the other hand, another study revealed a linear correlation between miR-221 expression and the pathological stage, lymph node involvement, Gleason Score, and biochemical recurrence (BCR) [121]. Yang et al. [122] confirmed that miR-221 and miR-222 are highly expressed in an androgen insensitive cell line (PC-3), and the experimental down-regulation of miR-221 or miR-222 inhibits migration and increases apoptosis in PC-3 cells. At the same time, the authors describe that the expression of SIRT1, a histone deacetylase, is increased in PCa cells after the inhibition of miR-221 and miR-222, suggesting that SIRT1 may play a suppressive role against the tumorigenic action of these miRNAs. To explore another possible mechanism of action of miR-221, a systematic biochemical and bioinformatical study has been performed [123]. It reveals two miR-221 targets, HECT domain E3 ubiquitin protein ligase 2 (*HECTD2*) and member RAS oncogene family (*RAB1A*). In this study, downregulation of HECTD2 affected androgen related transcription, and downregulation of HECTD2 and RAB1A altered the expression of many cell cycle genes and pathways, promoting tumor metastasis and leading to the development or maintenance of the CRPC phenotype.

#### 4.1.2. MiRNA Regulation of Androgen Signaling

Many investigations have been conducted for documenting the role of miRs in controlling the AR pathway. By using a miR library in 2011, Ostling et al. demonstrated the ability of 71 unique miRs (52 decreasing and 19 increasing) to influence the AR [124]. Since then several miRNAs have been described as having a role in the regulation of AR activity directly or through co-regulators [97,125].

MiR-205 is deregulated in PCa compared to benign prostate tissues, it is inversely associated to advanced disease and short life expectancy, and miR-205 levels exhibit a negative correlation to AR [126]. Moreover, miR-205 was also found to be lower in CRPC patients in comparison with men who had not initiated ADT. Hagman et al. [126] reported that mir-205 directly targets AR and reduces both AR transcript and proteins. The role of miR-205 is not only related to AR, but it has been found that this miRNA can regulate several genes. Some of these genes (*IL-8* and *EDN1*) are responsible for improving the expression of the AR, and others are involved in the MAPK/ERK, mTOR, and IL-6 signaling pathways [97].

MiR-34 family includes three miRNAs that have been previously reported to suppress tumorigenesis by different mechanisms, including modulation of cell cycle, epithelial to mesenchymal transition, or metastasis [127]. In PCa, all miR-34 family members are downregulated, and the expression of miR-34a or miR-34c correlates with the tumor grade, advanced disease, and life expectancy [128,129]. This down-regulation has been linked to several mechanisms such as methylation of the CpG islands in the promoter region of this miRNAs, regulation by p53 in response to DNA stress, and a mechanism involving the p38- MAPK/MK2 pathway [129,130,131,132]. As reported in the study by Ostling et al., in PCa cells a statistically significant inverse association exists between miR-34a and AR [124]. Recently, Fang et al. [133] demonstrated that the long non-coding RNA PlncRNA-1, known to be enhanced by AR, can preserve AR from miR-34c-mediated suppression in PCa cells. According to the theory of competing endogenous RNAs, some kind of RNAs may “titrate” other ribonucleic acids such as miRNAs [133].

LET7 levels are frequently decreased in human cancers [134,135]. The most important and well known targets of this miRNA are the oncogenes *RAS* and *MYC* [136,137]. A work by Nadiminty et al. explain that LET7c determines PCa tumor suppression through AR, and this mechanism is linked to the ability of this tumor-suppressing miRNA to target *c-MYC*, a molecule required for the correct transcription of AR [138]. In detail, the same group also found that LET7c reduces AR activity and decreases growth of C4-2B cells, and it can be attributable to the association of this miRNA with c-MYC 3′-UTR and the subsequent reduction of AR transcription [138]. These results are corroborated by other studies. Gao et al. [139] reported that the suppression of the AR and c-MYC diminishes PCa cell proliferation, but at the same time, an ectopic overexpression of c-MYC mitigates the tumor progression due to AR suppression, supporting an intense molecular relation between the AR and c-MYC.

Not only do miRNAs have direct effects, but they can also use other pathways to control androgen signaling. Two of these pathways are mediated by the ERBB-2 and PI3K/AKT. The tyrosine receptor ERBB-2 is often elevated in PCa, whereas the activation of PI3K/AKT signaling is linked to proliferation, metastasis, apoptosis resistance and angiogenesis in PCa [140]. A work by Epis et al. demonstrates that the *ERBB-2* mRNA 3′-UTR contains two specific miR-331-3p target sites and that miR-331-3p suppresses ERBB-2 expression at both the transcript and protein levels. MiR-331-3p expression was found to be lower in ERBB-2 overexpressing PCa tissue compared to normal adjacent tissue. The same group also explained that miR-331-3p is involved in the downstream PI3K/AKT signaling in multiple PCa cell lines. Interestingly, it has been shown that miR-331-3p acts specifically to decrease PSA promoter activity and PSA levels without reducing AR expression [140].

MiR-488* directly targets AR by targeting the AR in 3′-UTR. MiR-488* down-regulates AR protein expression in both androgen-sensitive and insensitive PCa cells, inhibiting cellular growth and increasing apoptosis as observed after the transfection of miR-488* [141].

MiR-17-5p has been shown to target PCAF, a coactivator of AR, and to support PCa development [142]. The authors found that the overexpression of PCAF in PCa cells is inversely associated with miR-17-5p levels, suggesting that low levels of miR-17-5p can enhance AR signaling in PCa cells indirectly by modulating PCAF expression. Moreover, circulating miRNAs of the miR-17 family have been recently associated with a reduction of PSA levels and overall survival in CRPC patients [143].

MiR-124 has been described as a tumor suppressor miRNA in several cancer types including PCa [144,145,146]. In accordance with its role in many biological processes, different authors examined the mechanism of action of miR-124. As reported in a recent review [97], the reduction of miR-124 levels in PCa cells is due to hypermethylation of the promoter. As a consequence of this event, both cell lines or clinical prostate samples showed an elevation in AR expression. Mechanistically, the presence of the miR-124-binding site in the AR 3′-UTR seems to explain the reason why miR-124 is involved in the negative regulation of the AR [147]. Moreover, Shi et al. reported that miR-124 can induce the upregulation of p53, causing cell death and apoptosis in AR-positive PCa cells [147].

The same authors propose an explanation of this phenomenon. The upregulation of p53 may in part be due to the capacity of miR-124 to inhibit the AR/miR-125b signaling pathway or by targeting the 3′-UTR of the high mobility group A (*HMGA*) gene which, as previously reported, can inactivate p53 [147]. A recent study shows that miR-124 can inhibit AR expression and suppress PCa cells proliferation and, on the other hand, that miR-124 is an androgen/AR responsive gene [148].

**Figure 3:**
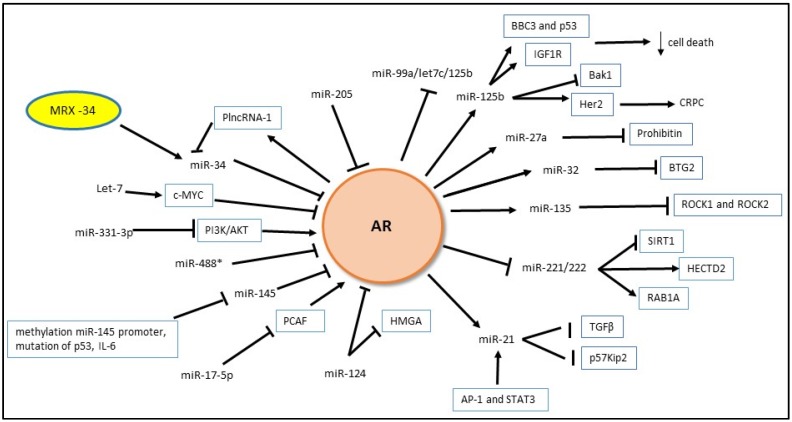
Mutual regulatory model of miRNAs and androgen receptor (AR). The graphic also shows MRX-34, the first miRNA based therapy for cancer. BBC3: Bcl-2-binding component 3; IGF1R: Insulin-like growth factor 1; Bak1: Bcl-2 homologous antagonist/killer 1; HER2: human epidermal growth factor receptor 2; p57Kip2: cyclin-dependent kinase inhibitor; AP-1: activator protein 1; STAT3: signal transducer and activator of transcription 3; HMGA: high mobility group A gene; PCAF: p300/cAMP-response element-binding protein associated factor; PI3K: phosphatidylinositol-3-kinases; PlncRNA-1: prostate cancer-up-regulated long noncoding RNA 1.

Finally, in the same class of small ncRNA we can include miR-145. MiR-145 has consistently been found to be downregulated in several types of cancer, including PCa [149,150], and it is inversely correlated with metastasis, survival and ADT response [151]. The reason for its downregulation is not completely clear. It could be due to the methylation of the miR-145 promoter, to the mutation of p53 that is a transcriptional activator of miR-145, or to the effect of IL-6 [151]. Larne et al. [151] theorized that miR-145 may determine a reduction of the AR and its target genes, *PSA* and *TMPRSS2*, at both transcription and protein levels by direct binding because the AR 3′-UTR contains a predicted miR-145 binding site. Moreover, using clinical prostate specimens the authors confirmed the same promising results, suggesting a future role of this miRNA as a novel therapeutic intervention. Our findings regarding the role of miRNAs in AR transcriptional activity are summarized in Figure 3.

### 4.2. Long Non Coding RNA and AR

Given the growing body of evidence documenting the role of long non coding RNA (lncRNA) in controlling various biological processes or having a central role in various cancers [152,153,154], it is reasonable to assume that lncRNAs may have a significant role in PCa as well. Several investigations in PCa suggest that specific lncRNAs can modulate AR activity through various mechanisms [97].

In 2000, Srikantan et al. [155] characterized the prostate cancer gene expression marker 1 (PCGEM1). PCGEM1 is overexpressed in more than half of PCa tissues [156], and its upregulation has been associated with high-risk PCa [157]. Moreover, the ectopic expression of PCGEM1 may be a cause of resistance to doxorubicin-induced apoptosis [158], and this can explain why a gene expression analysis found its levels upregulated in CRPC [159]. The prostate cancer noncoding RNA1 (PRNCR1) is transcribed from the “gene desert” region of chromosome 8q24. It is a 13 kb intron less lncRNA, and although the role of PRNCR1 is not well known, its knockdown reportedly inhibits cell viability [160]. Several works confirm that PCGEM1 is an androgen-regulated prostate-specific gene [155,161] and that PCGEM1 [162] as well as PRNCR1 [160] are involved in prostate carcinogenesis through AR activation.

An elegant study performed by Yang et al. [156] discovered a particular chromatin mechanism for AR transactivation mediated by PRNCR1 and PCGEM1. The authors explain that binding of PRNCR1 to the AR enhancer region and its association with DOT1L is fundamental for the enrollment of PCGEM1. As reported in the article, PCGEM1 needs the recruitment of Pygo2 to form a selective looping of the enhancer region in order to induce transcription of the target genes. Moreover, the authors state that PRNCR1 and PCGEM1 are indispensable for the activation of both truncated and full-length AR. Confirming these results, the knockdown of these lncRNAs in the CRPC cell line strongly suppresses the growth of the cancer in a xenograft model [156].

Nevertheless, the efficacy of these findings has been questioned. In fact, Prensner et al. [163] disagreed with these reports because they found that only PCGEM1 is associated with PCa. Moreover, using a large cohort of high-risk PCa patients, they showed the lack of an association of these lncRNAs with poor disease outcomes.

Recently, Ho et al. [164] described a new mechanism through which PCGEM1 can regulate AR expression in CRPC. They demonstrate that androgen deprivation induces the elevation of PCGEM1 through p54/nrb (engaged in RNA splicing and gene regulation) leading to expression of the splice variant AR3 and castration resistance disease.

The lncRNA PCA3, one of the most important prostate-specific genes, has been extensively studied as a tumor biomarker [165] due to its specific expression in both PCa and high-grade prostatic intraepithelial neoplasia [166]. PCA3 has been demonstrated to have a role in the regulation of AR signaling. Several experiments silencing PCA3 showed a reduction of AR target genes and a higher number of cells in the sub G0/G1 phase of the cell cycle [167]. Lemos et al. recently explained that PCA3 can be considered a significant marker to detect the “epithelial to mesenchymal transition” process [168].

Another lncRNA named CTBP1 antisense (CTBP1-AS) has been identified as a promoter of the AR transcriptional activity [169]. To explore the function of CTBP1, the authors use an antisense non-coding RNA. Thanks to this, it has been shown that CTBP1-AS works by repressing CTBP1 in two different scenarios. Firstly, CTBP1-AS acts with the RNA-binding transcriptional repressor PSF to recruit the HDAC–Sin3A complexes to CTBP1 promoter in cis with the loss of activating histone marks. Secondly, in the trans-regulatory pathway, CTBP1-AS also enhances PSF complexes to the regulatory regions of target genes, leading to the transcriptional repression of suppressive genes [169]. Despite the fact that its mechanism of action has been elucidated, we have opposing results regarding the effective levels of CTBP-1 in PCa. Takayama et al. [169] revealed the suppressive role of CTBP1 in AR-positive PCa cells, but there is another work describing not only that CTBP1 is upregulated in metastatic PCa but also that CTBP1 has a sort of stimulatory effect in PCa cells [170]. As suggested by the authors, this debate can be solved by analyzing tumor samples or cell lines used in these works. In fact, the experiments of Wang et al. were performed predominantly in AR-negative cells while Takayama et al. showed that CTBP1 exerts tumor suppressive effects in AR-positive PCa cell lines.

Cui et al. [171] firstly demonstrated that the expression of PlncRNA-1 is significantly higher in PCa cells compared to normal cells but also, more interestingly, that PlncRNA-1 silencing decreases AR mRNA- and AR-related genes. The authors give the same results in both androgen-dependent (LNCaP) and androgen-independent cell lines (LNCaP-AI). As above mentioned, the same group recently discovered that PlncRNA-1 can deregulate the expression of miR-34c and miR-297. At the same time, these two miRNAs have the ability to reduce PlncRNA-1 expression, creating a reciprocal inhibitory feedback loop [133].

The lncRNA HOTAIR is a 2.2-kb-long transcript localized to the boundaries of the HOXC gene cluster [172]. Tsai et al. [173] elucidated the role of HOTAIR as a scaffold protein that interacts at the 5′ domain with PRC2 and at the 3′ domain with the LSD1/CoREST/REST complex. This allows the concomitant methylation of H3K27 and the demethylation of H3K4 [173]. Zhang et al. [172] recently investigated the role of HOTAIR interacting with the AR. They discovered high levels of HOTAIR after ADT and further confirmed that its knockdown decreased cell proliferation. In the same work, one of the possible mechanisms underlying the effect of this lncRNA has been explained. HOTAIR seems to limit the AR ubiquitination and degradation, reducing the interaction between AR and the E3 ubiquitin ligase MDM2, and this can explain why the overexpression of HOTAIR can led to a CRPC condition [172].

Prostate cancer transcript-associated 18 (PCAT18) is a lncRNA reported to be prostate specific and up-regulated in PCa compared to other tumors [174]. An RNA sequencing on paired metastatic/non-metastatic PCa xenografts derived from clinical specimens showed the upregulation of PCAT18 in metastatic PCa. Furthermore, the same group discovered not only that AR can improve PCAT18 overexpression but that this lncRNA can be involved in PCa cell proliferation, cell migration and cell invasion [174].

Another novel lncRNA, PCAT29, was recently discovered, and its relationship to the AR explained. Malik et al. [175] described a different behavior of this lncRNA in presence or in absence of androgens. Specifically, PCAT29 is suppressed by dihydrotestosterone (DHT) and increased after ADT. Low or repressed levels of PCAT29 show an improvement in proliferation and migration of PCa cells; whereas PCAT29 overexpression confers the opposite effect and attenuates growth and metastasis of prostate tumors [175]. Moreover, Sakurai et al. [176] proposed a mechanism of regulation for this lncRNA based on an equilibrium between different molecules. In androgen-dependent cells, androgen stimulates AR to bind to the PCAT29 locus suppressing its expression. On the contrary, FOXA1 and NKX3-1 can balance the effect mediated by AR and prevent the repression of PCAT29. Interestingly, in castration resistant cells low levels of FOXA1 and NKX3-1 together with an anomalous activation of AR determine the decrease of PCAT29 [176].

## 5. Novel PCa Biomarkers

Prostate specific antigen (PSA) is the most important screening technique used for PCa diagnosis and tumor monitoring. Despite being organ specific, PSA is not cancer specific, and its level changes in the presence of several conditions such as prostatitis, hyperplasia, prostate biopsies and surgeries [177]. All of these pitfalls may determine over-diagnosis and over-treatment especially for low or very low-risk PCa patients [178,179,180]. Despite the fact that in the last decade innumerable molecules have been discovered, the inconsistency of some findings, the difficulty of reproducibility, and the lack of clinical studies with a significant number of patients can explain the reason why only a very small number of these markers have been used in clinical practice.

The epigenetic alterations in PCa, as in part described above, can provide effective biomarkers for early detection and cancer relapse, for prognosis and, finally, to predict then response to specific therapies [181]. In this section, we present the epigenetic biomarkers with a consistent role and use in clinical practice.

### 5.1. Epigenetic Signature as Biomarkers

Several reviews report the role of the DNA methylation asset as a biomarker for PCa detection/diagnosis or prognosis and response to therapy [181,182].

One of the most frequent epigenetic alterations in PCa is the aberrant promoter methylation of the glutathione S-transferase pi 1 (*GSTP1*) gene. In fact, *GSTP1* has been considered one of the most promising candidates for a DNA methylation biomarker because it appears in more than 90% of cases [183].

In 2011, Wu et al. [184] published a meta-analysis on GSTP1 methylation in body fluids. The authors highlight an excellent specificity (86.8%–100%) but a lower sensitivity in urine (18.8%–83.2%) and serum or plasma (13.0%–71.9%) samples. These findings suggest the possible role of GSTP1 methylation as a biomarker for PCa diagnosis [185]. In 2014, a review by Strand et al. [186] studied the ability of GSTPI methylation as a biomarker for disease prognosis. In this case the authors did not find strong evidence for the use of this gene in cancer tissues for predicting early disease outcomes [185].

In order to enhance the predictive power of this biomarker, several gene panels have been studied. The combination of GSTP1 with other DNA methylation biomarkers showed an improvement in the detection rate (86% for urine and 42%–47% for serum) [181].

Moreover, the combination of the methylation pattern of three genes (*GSTP1*, *APC*, and *RARB2*) has been evaluated in a prospective study named ProCaM [187]. The test performed on urine samples collected after DRE presented a higher predictive accuracy than simply using PSA and clinical characteristics [188].

A methylation marker genetic test, ConfirmMDx (MDxHealth, Inc., Irvine, CA, USA), is a tissue-based assay that studies the epigenetic alteration surrounding the tumor lesions. This test identifies the methylation pattern of three genes (*GSTP1*, *APC*, and *RASSF1*) in men with a low risk for disease after a negative biopsy. ConfirmMDx, after a validation in a European and a US population, achieved a negative predictive value of 88%–90% [189,190]. Furthermore, the 2016 Clinical Guide of the National Comprehensive Cancer Network [191] recommended this test for the early detection of PCa in patients with an elevated PSA and prior negative biopsy.

To explore the biological role of DNA methylation alterations and to understand the utility of these epigenetic modifications as future biomarkers or as therapeutic targets, Aryee et al. [192] used a genome-scale analysis of DNA methylation among metastatic PCa patients. Although a consistent inter-individual heterogeneity in DNA methylation alterations was found, the authors showed that the methylation signatures are preserved in each patients’ metastases. This interesting intra-individual homogeneity is a promising finding in the development of personalized treatments against all lethal metastatic PCa cell clones [192].

### 5.2. Long Non-Coding RNA as Biomarkers

Prostate cancer antigen 3 (PCA3) is a prostate cancer-specific antigen mapped to chromosome 9q21-22 [193]. It is a lncRNA of unknown function identified by Bussemakers et al. in 1999 [194]. More than 95% of PCa specimens show PCA3 over-expression [194], and in addition to cancer tissue, PCA3 transcripts have also been identified in urine samples of patients with benign enlargement and malignant disease of the prostate [165]. The Progensa PCA3 test (Hologic Gen-Probe, Marlborough, MA, USA), an in vitro amplification test, attained Conformiteé Européenne (CE) in 2006, and it was approved by the US FDA in 2012 for use in men older than 50 years old with one or more negative biopsies [195].

The PCA3-test involves collection of a urine sample after DRE to mobilize prostatic cells. The PCA3 score is a mathematical operation, and it can be acquired by dividing PCA3 RNA by PSA RNA levels in order to normalize PCA3 signals.

Despite these suggestive findings, the best cut-off to use is still controversial. A urine PCA3 score more of than 35 has been linked with a sensitivity comprised between 47%–57% and a specificity around 70% [196,197]. A recent meta-analysis by Lu et al. describes a sensitivity and specificity of 72% and 53%, respectively, with a PCA3 score cut-off of 20 [198]. Crawford et al. in a large multicenter study showed that a cut-off of 35 is correlated with a large number of false negatives, even though it can reduce the number of re-biopsies by 77% [199]. Controversial results have been reported regarding the relationship between PCA3 score and aggressive features. Some studies describe a correlation with the Gleason Score, the tumor volume, and extracapsular extension [200,201], but others didn’t find any correlation with the aggressiveness of the tumor [202,203]. Despite these conflicting results, PCA3 score has been considered a more specific indicator with a better predictive value than the PSA [204].

In 2013, GenomeDx Biosciences (Vancouver, BC, Canada) and Mayo Clinic (Rochester, MN, USA) co-developed and validated a tissue-based genomic classifier that is able to evaluate the risk of developing clinical metastases at 5 years postoperatively named Decipher [205]. The authors used a high-density transcriptome-wide microarray to assess the expression of over 1.4 million markers including protein-coding genes and ncRNAs in 545 PCa patients samples including 213 who experienced early metastasis [205]. This test, whose result is expressed as a continuous risk score ranging from 0 to 1, is based on 22 RNA biomarkers related to cell proliferation, differentiation, motility, immune modulation and AR signaling. Decipher has been studied by several groups with varying cohorts of patients [206]. Karnes et al. [207] evaluated the prognostic role of Decipher in 219 high-risk PCa patients with a median follow up of 6.7 years after surgery. On multivariable analyses, higher decipher scores resulted in the highest prognostic predictor of metastasis with an area under the curve (AUC) of 0.79. 85 high-risk patients with PSA failure after radical prostatectomy (RP) were evaluated by Ross et al. in 2014 [208]. The genomic classifier showed an AUC of 0.82 compared to 0.64 of Gleason Score and 0.69 of PSA doubling time. The prognostic value of Decipher has also been studied in PCa patients undergoing adjuvant or salvage radiation therapy following RP. Den et al. [209] demonstrated that the AUC of this genomic test is 0.78 and 0.80 to predict BCR and metastasis, respectively, in a cohort of 139 patients treated by RP and adjuvant radiotherapy. Patients with a higher genomic score mainly benefit from this adjuvant treatment. With a median follow-up of 10 years, the same group followed 188 patients from two different institutions treated with RP and adjuvant or salvage radiotherapy [210]. Decipher predicts the occurrence of metastases on multivariable analyses and confirmed the previous results suggesting that adjuvant radiotherapy should be taken into consideration for PCa patients with high genomic score. Approved in the United States for patients with positive margins, pT3 disease, or PSA failure after surgery [211], Decipher could help physicians in the clinical decision making in order to improve accuracy in predicting patient outcomes.

### 5.3. MicroRNA as Biomarkers

Several studies suggest the use of miRNAs in the clinical setting and many reviews have been published about the argument [97,212,213]. The exploitation of different technological platforms, the examination of different samples (tissues, sera, urine), the retrospective design of many studies, the use of endogenous or exogenous controls, and the presence of contaminating non-neoplastic cells are all potential explanations for controversial results reported until now. Despite these obstacles, miRNAs have been reported to have a promising role as novel biomarkers in PCa. In the last few decades, many miRNAs profiles have been presented, but there is not a large consensus in the expression of a single signature in different groups. For this reason, many efforts have been undertaken to discover a panel of small RNAs in order to reduce the inter-individualities between several settings.

Larne et al. [149] focused their attention on a combination of four miRNAs. These four discriminatory miRNAs (miR-96-5p, miR-183-5p, miR-145-5p, and miR-221-5p), characterize the miR index quote (miQ). This test seems to predict PCa (AUC = 0.931) after a validation in four external cohorts. In addition, miQ was investigated to predict the manifestation of metastases (AUC = 0.827) and unfavorable disease behavior (AUC = 0.895).

As mentioned above, several miRNAs, regulated by or involved in AR transcriptional activity, are able to predict biochemical failure, clinical relapse, and castration resistant status. Interestingly, the overexpression of miR-21 has been reported in patients with castration-resistant disease [214], and it is reported to be an independent predictor of BCR in patients with a Gleason Score of 6 [215]. In the same way, miR-221 and miR-222 have been found to be upregulated in CRPC patients [216], and other studies demonstrate that miR-221 is also able to predict both recurrence and cancer related death [217,218].

## 6. Novel Treatments

Androgen deprivation therapy (ADT) is the principle treatment for advanced PCa and induces remission in 80%–90% of patients [219]. Despite an initial response, cancer cells are able to escape, and they subsequently continue to proliferate. This condition is termed castration resistant prostate cancer (CRPC), and it reportedly has a median overall survival rate of 23–37 months from the start of ADT [220]. The mechanisms governing the reactivation of AR despite castrated levels of testosterone have been widely studied. Although several alternative pathways have been observed and reported [221,222], the predominant mechanisms for cancer cell proliferation under deprivation conditions are due to reactivation, overexpression or mutation of the AR [223]. Therefore, therapies aimed to block the AR or to block the crosstalk of this steroid receptor with other molecular pathways are considered promising approaches to treat CRPCs. Here, we present three examples of how the regulation of AR epigenomic mechanisms could offer a novel therapeutic target to limit PCa proliferation.

### 6.1. Demethylase Inhibitor

Uncovering the locations of proteins throughout the genome helped physicians to understand the biology of both healthy and tumoral prostates. Chromatin immunoprecipitation (ChIP) followed by high-throughput DNA sequencing (ChIP-seq) is considered a novel technique to discover transcription factor binding sites, chromatin regulators, and the identification of genomic histone marks. Moreover, mapping the interactions of small molecules with chromatin, a technique named Chem-seq, has not only helped build the understanding of novel mechanisms underlying the biology of diseases but in discovering new specific treatments [224].

Chem-seq, ChIP-seq, and RNA-seq methods were used to evaluate the role of a small molecule, termed SD70, originally recognized as an inhibitor of DHT and chromosomal translocations events in PCa [225]. The 8-hydroxyquinoline domain of SD70 has been found to be similar to other molecules considered as competitive inhibitors of the histone demethylase KDM4 family and in particular KDM4C [226]. A biotinylated derivative of SD70 (B-SD70) has been observed as having the ability to bind the AR regulatory enhancers in an androgen-dependent manner. Further experiments show that SD70 was able to suppress DHT-regulated gene transcriptions in androgen dependent and independent cell lines, but at the same time, AR localization was not altered. As a consequence of its structure and analogy with other histone demethylase inhibitors, SD70 was found to inhibit the demethylase activity of KDM4C (Figure 1). KDM4C, as aforementioned above, plays a role in AR transcriptional program, mainly regulating the histone H3K9me3/me2 demethylase activity [50,227,228]. Using a Chem-seq assay on AR target gene enhancers, the authors reveal that KDM4C is located on the same gene enhancers that are co‑occupied by B-SD70.

RNA-seq analysis in KDM4C knockdown cells confirmed the central role of KDM4C on AR target-gene regulation. Moreover, a Chip-seq in androgen dependent cells revealed that SD70 represses the methylation activity of KDM4C at the AR-regulated enhancers [225]. In consideration of the fact that “in vivo” experiments with a xenograft model unveiled a conspicuous inhibitor effect of SD70 on tumor cell growth without any particular toxicity, SD70 should be considered a potential candidate therapy in PCa patients.

### 6.2. Deacetylase Inhibitor

Histone deacetylase inhibitors (HDACi) are a group of molecules with anticancer activity against hematologic and solid tumors [229]. Different classes of HDAC, as previously described, modulate the acetylation profile of numerous genes including AR. Several studies have reported that HDAC inhibitors, such as trichostatin A (TSA), suberoylanilide hydroxamic acid (SAHA), and valproic acid, may reduce AR expression [230,231,232], but their mechanisms of action are not completely clear. One of them is undoubtedly correlated to the heat shock protein-90 (HSP90). HSP90 is a chaperone protein indispensable for molecular stability and the right folding and function of steroid hormone receptors such as the AR [233,234] (Figure 2).

A recent review [235] extensively reported the activity of several HDACi as novel therapeutic options in CRPC but here we might focus our attention on the efficacy of HDACi which mainly affect the HSP90-AR signaling.

Romidepsin is a cyclic depsipeptide, enhancing the acetylation of HSP90, that reportedly interferes with the correct folding of AR determining its degradation [236]. Despite these encouraging pre-clinical data and two phase I clinical trials that did not show a particular toxicity, a phase II study (NCT00106418) unveiled a very low clinical activity in 35 metastatic CRPC patients. In particular, only two enrolled patients displayed a PSA reduction more than 50% in a period of time longer than 6 months. Moreover, a substantial proportion of patients (31%) interrupted the trial due to several toxic effects [237]. As suggested by the same authors, these data do not support the use of single-agent romidepsin in unselected CRPC patients.

Panobinostat is a cinnamic hydroxamic acid class molecule with an HDACi activity. In vivo studies in AR-positive PCa cell lines showed a significant degradation of the AR mediated by the acetylation and subsequent inhibition of the HSP90 chaperone function [238]. In 2010, Rathkopf et al. reported the first results of a phase I clinical trial (NCT00663832) of oral panobinostat versus oral panobinostat plus docetaxel in patients with advanced disease. Despite the fact that all patients being solely treated with panobinostat displayed a clinical progression, 63% of patients treated with a combination therapy exhibited a biochemical response greater than 50% [238]. The same group examined the effect of intravenous panobinostat in a phase II trial (NCT00667862). Of the 35 enrolled patients, none of them exhibited a significant PSA reduction [239]. Again, despite promising preclinical data and a strong scientific rationale, panobinostat has not shown a sufficient level of clinical activity as a single agent in metastatic patients.

### 6.3. Non Coding RNA Therapy

In the last decade, several findings have documented the role of miRNAs as new oncogenes or tumor suppressor genes, thus supporting their use as therapeutic tools. Artificial miRNA mimics and inhibitors are considered a good way in which to “block or boost” the production of several proteins [240]. MiRNA mimics have been used to reintroduce tumor suppressor miRNAs, and miRNA inhibitors serve to reduce the levels of oncogenic miRNAs. Interesting results from preclinical studies using mouse models demonstrate the possible therapeutic application of miRNA mimics in PCa [241]. The bi-univocal correlation between p53, one of the most important tumor suppressor genes, and miR-34 highlighted the role of this miRNA as an encouraging therapy for cancer [242]. MiR-34a seems to be a promising target in PCa because “in vivo” studies proved that its reintroduction decreases the growth of prostate xenografts [243]. In April 2013, a liposome-formulated miRNA34a mimic (MRX34), sponsored by Mirna Therapeutics (Austin, TX, USA), was tested in a phase I clinical trial (NCT01829971) [244] (Figure 3). This was the first attempt to use a miRNA as an innovative therapy for cancer.

## 7. Conclusions

The androgen receptor is the central regulator of nominal and tumor prostate biology. Prostate carcinogenesis is a complex event due to genetic mutations and epigenetic alterations. In the past decade, the role of epigenetic regulation has become evident, and considerable progress has been made defining its role in the onset and progression of prostate cancer. In this review we focused our attention mainly on the AR epigenetic alterations. A better understanding of AR transcriptional pathway is indispensable to develop diagnostic and therapeutic procedures exploiting these epigenetic changes. Although PSA remains the prevalent test for prostate cancer screening and prognosis, the new generation of biomarkers can help physicians in their clinical decisions. The PCA3 test is widely used in clinical practice but chromatin remodeling marks and miRNA panels, as well as genomic tests, are becoming new promising predictive tools. New technologies for global epigenomic analyses and integration with genomic and transcriptomic data are extending our knowledge on prostate tumorigenesis. A new approach named “Chem-seq” permitted us to uncover the site and the mechanism of action of a small molecule named SD70. The demethylase SD70, targeting a key regulator of AR function, is effective in CRPC cells in combination with current therapies. Furthermore, the optimization of the stability of miRNAs and the improvement of the efficacy of HDAC inhibitors are also challenges for the future treatment of prostate cancer. Knowing the specific molecular mechanisms underlying tumors will be desiderable for the identification of more effective approaches allowing to personalize therapy.
